# The Impact of Physical Activity at School on Children’s Body Mass during 2 Years of Observation

**DOI:** 10.3390/ijerph19063287

**Published:** 2022-03-10

**Authors:** Katarzyna Ługowska, Wojciech Kolanowski, Joanna Trafialek

**Affiliations:** 1 Faculty of Medical and Health Sciences, Siedlce University, 08-110 Siedlce, Poland; katarzyna.lugowska.zdoz@uph.edu.pl; 2 Faculty of Health Sciences, Medical University of Lublin, 20-400 Lublin, Poland; 3 Institute of Human Nutrition Sciences, Warsaw University of Life Sciences, 02-787 Warsaw, Poland; joanna_trafialek@sggw.edu.pl

**Keywords:** body mass index, children, obesity, overweight, physical activity

## Abstract

(1) Background: Children’s overweight and obesity are a growing public health problem. The aim of this study was to assess the influence of physical activity (PA) at school on body mass of children aged 10–12 during 2 years of observation. (2) Methods: Primary school children (*n* = 245, 48% girls and 52% boys) took part in the study. Children were divided in two groups, (1) of standard PA and (2) of elevated PA at school corresponding to 4 and 10 h of physical education lessons (PE) a week, respectively. Weight, height, and body mass index (BMI) were measured starting from the 4th grade and ending at the 6th grade of school. (3) Results: The number of children with excessive body weight (overweight and obese) increased by ¼ in children of standard PA while slightly decreased in children of elevated PA. Many more children of elevated PA changed body mass category from overweight to healthy weight than those of standard PA. Girls, especially of standard PA, had more often excessive body weight compared to boys. (4) Conclusions: Increasing time of physical activity at school by elevation of the number of PE lessons favorably affects the body mass of children.

## 1. Introduction

Overweight and obesity in children and teenagers are one of the most serious public health challenges in the 21st century [1]. The prevalence of overweight and obesity is increasing at an alarming pace and the problem affects all countries, regardless of their affluence [2,3]. The World Health Organization (WHO) has estimated that the problem of excessive body mass affects over 340 million children worldwide. In many countries, over 20% children are overweight or obese [1,4,5,6]. The WHO has adopted the “No increase in childhood overweight” project as one of the most important global nutrition targets [7]. In Poland as well, one in five school-age children are overweight or obese [8,9,10,11]. Commonly, children have access to computers, smartphones, and other mobile devices, increasing the amount of so-called screen time and reducing time of physical activity [11]. Moreover, the COVID-19 pandemic and the periodic lockdowns and online classes contribute to a further reduction in the physical activity of children, an increase in sedentary behavior and body weight [12,13].

Primary school is the first place where children acquire systematic knowledge about physical activity (PA) and health through physical education lessons (PE). PA is an important element of a healthy lifestyle [14,15]. However, a small percentage of school-age children and teenagers meet the requirements concerning daily PA level [16,17]. In Poland, prior to the COVID-19 pandemic, only 17% of children and teenagers undertook moderate PA [18,19]. An elevated level of PA among children leads to a reduction in the risk of overweight and obesity, and improves body composition, health, and fitness [20,21,22]. It is estimated that, on average, 80% of school-age children do not achieve the recommended PA level [23]. WHO recommends that children and teenagers should undertake at least 60 min of PA of moderate or high intensity every day [24]. Additionally, sedentary lifestyle should be reduced, including screen time such as use of computers smartphones, or watching TV, and regular PA undertaken instead [25,26].

The aim of this study was to assess the influence of the time of physical activity at school on body mass of children aged 10–12 during 2 years of observation.

## 2. Materials and Methods

### 2.1. Participants

The study was conducted in six primary schools in Siedlce (Poland), which had parallel classes with a standard and elevated number of PE lessons, so-called general education classes (GC) and sport classes (SC), respectively. In Poland, education with an elevated number of PE lessons (SC) in regular primary schools began with fourth grade school (children age approximately 10 years). The mandatory number of PE lessons for SC and GC was 10 and 4 h per week, respectively. PE lessons consisted of physical activity in the form of fitness exercises and team games. All schools’ management, children, and their parents expressed their consent to participate in the study. All of them were informed about the objective of the study and the confidential nature of the results. An approval of the Ethics Committee at the Siedlce University (no. 2/2016) was obtained for the study.

The study was conducted in the same way in all schools. The criteria for exclusion of particular results from the analysis were as follows: absence on one of the measurement days, inappropriate age of the child, failure to pass to the next grade, lack of the child’s consent on the measurement, diagnosed chronic diseases, acute diseases of the musculoskeletal or nervous systems, and orthopedic problems. However, none of the children’s parents reported problems with diagnosed chronic diseases, diseases of the musculoskeletal or nervous systems. The studied classes did not belong to the integration units in which there could be children with health disorders that had an impact on the final results. In the case, when during the study a child was admitted to the GC or SC as a result of a transfer from another parallel class or from another school, their results were excluded from the final analysis. The results concerned the same group of children who participated in all measurement sessions during the entire study period.

### 2.2. Research Methodology

The study was an observational follow-up with a control group and concerned the assessment of BMI among children attending classes of different level of PA over a period of 2 years. The test group was children of elevated PA at school (SC), the control group was children of standard PA at school (GC). The study consisted of periodic measurements of body weight and height of the GC and SC children starting from the fourth grade of school (age approx. 10 years), i.e., the grade when the elevated number of PE lessons was introduced, until the beginning of the sixth grade of school (age approx. 12 years). The body mass index (BMI) was calculated and interpreted with reference to the BMI percentile grids. Based on this, body mass categories, i.e., obesity, overweight, healthy weight, and underweight, were determined.

The study was preceded by a pilot study carried out in May–June 2017 on a group of children (*n* = 43) aged on average 10.8 years who attended both the GC and the SC classes (GC *n* = 22; SC *n* = 21). The purpose of the pilot study was to check the adopted methodology, to assess the procedures and fluidity of the measurements. The methodology was properly planned and proceeded without any problems. The methods verified in the pilot study were accepted for the main study.

The main study was conducted from September 2017 until October 2019, i.e., from the beginning of the fourth grade until the beginning of the sixth grade. The interpretation of the results focused on an analysis of the variability of the body mass categories in relation to the class profile which reflects the number of PA lessons, as well as to the gender and age of children. The measurements were conducted by members of the research team in the presence of teachers at 5–6 month intervals. The first measurement session (initial) was completed between 1 and 31 September 2017, the second between 1 and 30 March 2018, the third between 1 and 31 September 2018, the fourth between 1 and 30 March 2019, and the fifth (final one) between 1 and 31 September 2019. 

In total, five measurement sessions were carried out and the results were systematically processed and analyzed in Microsoft Excel. Each child was assigned an identification number and the results were collected in GMON MDD software (Medizin & Service GmbH, Boettcherstr, Chemnitz, Germany) [27] that works with a measurement device.

### 2.3. Measurements

The anthropometric measurements of height (cm) and body weight (kg) were carried out in accordance with the standard procedures described by the International Society for the Advancement of Kinanthropometry (ISAK) [28]. All measurements were performed using uniform standard equipment. The height measurements were performed with the child standing in an upright position using a SECA 214 height measuring rod (stadiometer) with an accuracy of 1 cm. The body weight was measured using a Tanita SC-240MA (Tanita Cooperation, Tokyo, Japan) [29] device with an accuracy of 0.1 kg. The calculated BMI was compared to the percentile grids for the population of Polish children [30,31]. According to the national criteria, BMI ≥ 85th percentile was assumed as overweight, BMI ≥ 95th percentile as obesity, and BMI ≤ 10th percentile as underweight [30,31].

### 2.4. Statistical Analysis

The statistical analysis of the results was undertaken using a Microsoft Excel 19 (Microsoft, Intentional Software, Washington DC, USA) [32] spreadsheet and Statistica software (Stat Soft, Krakow, Poland) [33]. It was assumed that the level of statistical significance of α < 0.05, where *p* < 0.05, indicated significant differences, while values of *p* > 0.05 were interpreted as insignificant. The average values were calculated at the level of class profile (GC, SC), gender, and age. Student’s t-test was used to compare the BMI values at the class profile and gender. Additionally, a principal component analysis (PCA) was undertaken. The PCA used the average results of the initial and final measurement sessions for the BMI, and the number of physical education lessons equal to 4 and 10 h for the GC and SC, respectively.

## 3. Results

### 3.1. Description of the Group

In total, the study involved 245 children (48% girls—G; 52% boys—B). The numbers of children in terms of the class profile were similar (SC—*n* = 122; GC—*n* = 123). In classes with an elevated number of PA lessons (SC), the study covered 143 children (G—67; B—76). However, due to some failure to meet the criteria, approx. 15% of the children (*n* = 21) (G—9%; B—20%) were not included in the final analysis of the results. The most common reasons for such exclusion were a change in school or class (10%) and joining a class during the study (5%). Finally, the analysis covered 122 SC children, who attended all measurement sessions (G—61; B—61). In the case of the GC group, the study covered 161 children (G—75; B—86). Finally, the analysis covered 123 GC children, who attended all measurement sessions (G—57; B—66). Similar to the SC, the most common exclusion factors in the GC group were a change in school or class (13%) and joining a class during the study (10.5%).

### 3.2. Evaluation of the Anthropometric Indicators

#### 3.2.1. Height

The average heights of children in classes of both types were similar. At the start of the study, the average height for the entire group was 142.96 cm (GC—143.37 cm; SC—142.55 cm; *p* = 0.350). At the end of the study, the average height for the entire group was 156.25 cm (GC—156.69 cm; SC—155.81 cm; *p* = 0.400). On average, during the study, GC children had a slightly greater height than the SC ones (GC—150.12 cm; SC—149.19 cm). The greatest and smallest heights were observed in the GC (125.00 and 186.00 cm, respectively). No significant differences in the average height between the GC and the SC were observed. On average, during the study, the girls’ height increased by 14.01 cm (GC—14.30 cm; SC—13.72 cm; *p* = 0.940) and the boys’ height increased by 12.64 cm (GC—12.47 cm; SC—12.82 cm; *p* = 0.240). The statistical analysis did not demonstrate any statistically significant differences between the class profile.

#### 3.2.2. Body Weight

Children attending the SC had slightly lower average body weight compared to the GC ones (SC—42.24 kg; GC—43.85 kg; *p* = 0.410). Generally, the average increase in the body weight during the study was 10.85 kg (GC—11.13 kg; SC—10.58 kg; *p* = 0.320). The differences between the GC and the SC were not significant.

The average increase in the body weight was 11.34 kg for girls (GC—11.68 kg; SC—11.01 kg; *p* = 0.990) and 10.41 kg for boys (GC—10.68 kg; SC—10.14 kg; *p* = 0.220). In general, girls’ body weight was slightly lower than boys’ (G—42.88 kg; B—43.13 kg). The differences between the GC and the SC boys’ and girls’ average body weights were not significant.

### 3.3. BMI

The BMI of the GC children ranged from 12.60 to 40.00 kg/m^2^, and that for the SC from 12.90 to 29.00 kg/m^2^. On average, a higher BMI was observed in the GC group. Over the entire study period, the BMI increased on average by 1.42 (GC—1.52 kg/m^2^; SC—1.32 kg/m^2^). However, the average BMI, for both the GC and the SC, were in the healthy range in each group. At the beginning of the study, the average BMI for the GC was 18.41 kg/m^2^, and for the SC 18.26 kg/m^2^ (*p* = 0.721). The final measurement session indicated an increase in the BMI to 19.93 kg/m^2^ for the GC and 19.58 kg/m^2^ for the SC (*p* = 0.460). There were no significant differences between the average BMI for the GC and the SC during the study.

In general, the SC girls had a higher average BMI value in the beginning of the study than the GC ones (GC—18.20 kg/m^2^; SC—18.37 kg/m^2^; *p* = 0.761). However, in the subsequent measurements, the GC girls had a higher average BMI value than the SC ones. At the final measurement, the average BMI for the GC girls was 19.82 kg/m^2^, and for SC girls—19.74 kg/m^2^ (*p* = 0.890).

The GC boys had a higher average BMI value at the beginning of the study than the SC ones (GC—18.60 kg/m^2^; SC—18.15 kg/m^2^; *p* = 0.501). However, similar to the girls, at the final measurement session, the GC boys had a higher average BMI value, which was equal to 20.03 kg/m^2^, compared to 19.42 kg/m^2^ for the SC ones (*p* = 0.401). It was also shown that the SC boys had a lower average BMI value compared to the GC ones. The average increase in the boys’ BMI over the study period was equal to 1.35 kg/m^2^ (GC—1.43 kg/m^2^; SC—1.27 kg/m^2^). The statistical analysis did not show any significant differences.

On average, during the study, a healthy range of body weight was identified for 65.90% of the children (GC—63.95%; SC—67.71%); underweight—for 8.32% (GC—7.96%; SC—8.68%); overweight—for 17.08% (GC—18.90%; SC—15.25%); and obesity—for 8.78% (GC—9.19%; SC—8.36%). Both healthy weight and underweight were more common in the SC group than the GC one (Table 1).

The highest percentage of underweight children was shown at the start of the study (grade 4, the average age of 10.27 years) and it concerned 10.17% of children (GC—9.69%; SC—10.66%; *p* = 0.720). The lowest percentage of underweight was shown after 1.5 years and concerned 6.05% of children (GC—5.54%; SC—6.55%; *p* = 0.460). The highest percentage of healthy weight was shown in grade 5 (the average age of 11.27 years) and concerned 66.71% of children (GC—65.39%; SC—68.03%; *p* = 0.350). The lowest percentage of healthy weight was shown in grade 6 (the average age of 12.26 years) and concerned 65.25% of children (GC—60.01%; SC—70.51%; *p* = 0.460). The highest percentage of overweight children was shown at the end of the study (grade 6, the average age of 12.26 years) and concerned 19.76% of children (GC—24.76%; SC—14.76%; *p* = 0.461). The lowest percentage of overweight was shown at the start of the study (grade 4, the average age of 10.27 years) and concerned 15.27% of children (GC—13.31%; SC—17.22%; *p* = 0.722). The highest percentage of obesity was shown in grade 5 (the average age of 11.90 years) and concerned 9.85% of children (GC—9.05%; SC—10.66%; *p* = 0.461).

Healthy body weight was shown more often in girls attending the SC than those attending the GC, while overweight was more common in the GC than the SC (Table 2). On average, during the study, healthy weight concerned 64.58% of the girls (GC—64.56%; SC—64.60%); underweight—7.39% (GC—5.61%, SC—9.17%); overweight 18.91% of the girls (GC—21.75%; SC—16.07%); and obesity 9.12% (GC—8.08%; SC—10.16%).

The BMI of the girls with healthy weight attending the GC was on average 17.18 kg/m^2^ in the initial measurement session and 18.16 kg/m^2^ in the final measurement session (an increase by 0.98 kg/m^2^, *p* = 0.008). The BMI of the girls with healthy weight attending the SC was higher than for the GC and was equal on average to 17.40 kg/m^2^ in the initial measurement session and 18.43 kg/m^2^ in the final one (an increase by 1.03 kg/m^2^, *p* = 0.002). Statistically significant differences were observed for girls with healthy weight between the initial and final measurement sessions.

Girls with excessive body weight (overweight and obese) attending the GC had on average a BMI equal to 22.18 kg/m^2^ in the initial measurement session and 23.79 kg/m^2^ in the final one (an increase by 1.61 kg/m^2^). For the SC ones it was 22.58 and 24.57 kg/m^2^, respectively (an increase by 1.99 kg/m^2^). Significant differences between the BMI for girls with excessive body weight attending the SC between the initial and final measurement sessions were shown (*p* = 0.000), while for the GC the differences were not significant (*p* = 0.260).

On average, the boys attending the SC had healthier body weight compared to the GC ones (Table 3). Over the entire study period, healthy weight concerned 67.08% of the boys (GC—63.34%; SC—70.82%); underweight—9.25% (GC—10.30%, SC—8.19%); overweight 15.25% (GC—16.06%; SC—14.43%); and obesity 8.43% (GC—10.30%; SC—6.56%). Healthy weight was more common among the SC boys than the GC ones, while overweight and obesity among the GC boys (*p* = 0.400).

Between the initial and final measurement sessions, the average BMI of SC boys increased from 14.06 to 14.66 kg/m^2^ (*p* = 0.058). In the GC, the BMI increased from 14.00 to 15.04 kg/m^2^. A statistical analysis showed significant differences between the initial and final measurements (*p* = 0.000).

The BMI of the GC boys with healthy weight was similar to that for the SC and in the initial and final measurement sessions was equal to 17.25 and 18.45 kg/m^2^, respectively (*p* = 0.007). While the BMI for the SC was equal to 17.37 and 18.47 kg/m^2^, respectively (*p* = 0.004).

The BMI of the GC boys with excessive body weight (overweight and obese) was on average 25.24 kg/m^2^, while at the final measurement session it was 25.55 kg/m^2^ (*p* = 0.848). For the SC overweight and obese boys, the average BMI was equal to 22.54 and 24.60 kg/m^2^, respectively (*p* = 0.014).

During the study, healthy weight and underweight were shown more often in boys than in girls. Underweight was observed on average in 10.30% of GC boys and 8.19% of SC ones, and in 5.61% of GC girls and 9.17% of SC ones. Healthy weight was shown on average in 67.08% of boys (GC—63.34%; SC—70.82%) and 64.58% of girls (GC—64.56%; SC—64.60%). Overweight was shown on average in 18.91% of girls (GC—21.75%; SC—16.07%) and 15.25% of boys (GC—16.06%; SC—14.43%). Obesity was shown on average in 9.12% of girls (GC—8.08%; SC—10.16%) and 8.43% of boys (GC—10.30%; SC—6.56%).

### 3.4. Analysis of BMI Variability during the Study

**Figure 1:**
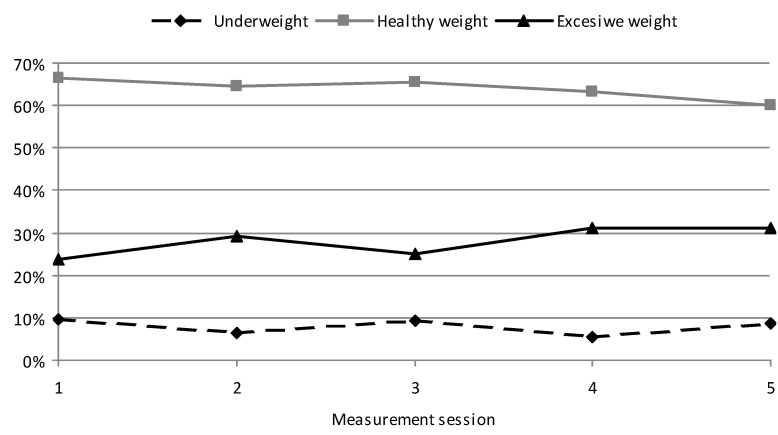
Changes in the percentage of excessive body weight, healthy weight, and underweight in children of standard PA (GC) during the course of the study.

During the study, an increasing percentage of children with excessive body weight (overweight and obese) was observed in the GC, from the initial 23.76% to 31.18% in the final measurement session (*p* = 0.007). A decreasing percentage of underweight children was shown, from 9.69% to 8.81% (*p* = 0.001). The percentage of GC children with healthy weight decreased from 66.55% to 60.01% (*p* = 0.000) (Figure 1). The highest percentage of underweight GC children was shown at the start of the study (9.69%), while the lowest one was shown in the 4th measurement session for the average age of 11.0 years (5.45%). The number of children with excessive body weight increased with age, while healthy weight decreased. The highest percentage of GC children with healthy weight was shown at the start of the study (66.55%) and the lowest one at the final measurement session (60.01%).

**Figure 2:**
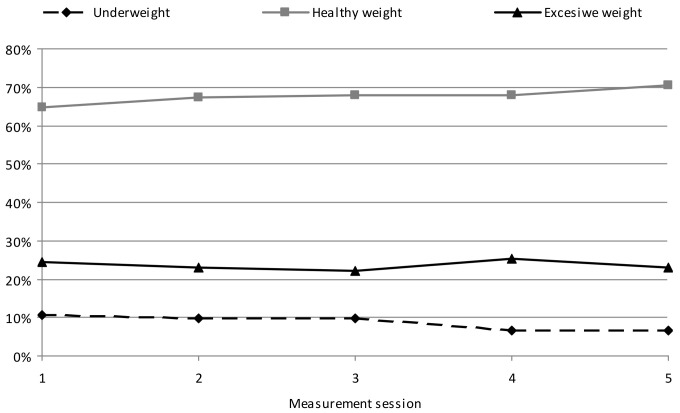
Changes in the percentage of excessive body weight, healthy weight, and underweight in children of elevated PA (SC) during the course of the study.

Oppositely to the GC, in the SC children a decrease in the percentage of children with excessive body weight was shown during the study, from 24.59% in the initial measurement session to 22.94% in the final one (*p* = 0.000). The percentage of underweight children decreased from 10.66% to 6.55% (*p* = 0.012) (Figure 2). A growing percentage of the SC children with healthy weight from 64.75% to 70.51% was shown (an increase by 5.76%) (*p* = 0.000). The highest percentage of underweight SC children was shown at the start of the study (10.66%), while the lowest one in the final measurement session (6.55%). The percentage of children with excessive body weight was the lowest in the 4th measurement session (25.42%) and the lowest in the 3rd measurement session (22.14%); however, these differences were not significant.

Generally, during the study an increase in the number of the GC boys and girls with excessive body weight and a decrease in the SC ones were shown. At the start of the study, 15.29% of the children were overweight, including 13.31% of the GC and 17.22% of the SC ones. At the end of the study, nearly 20% of the children were overweight, including 24.76% of the GC and 14.76% of the SC ones. At the start of the study (fourth grade), approx. 65% of the children had a healthy weight. However, the final measurements session (sixth grade) demonstrated a smaller percentage of healthy weight children in the GC (60.01%) and significantly higher in the SC ones (70.50%).

**Figure 3:**
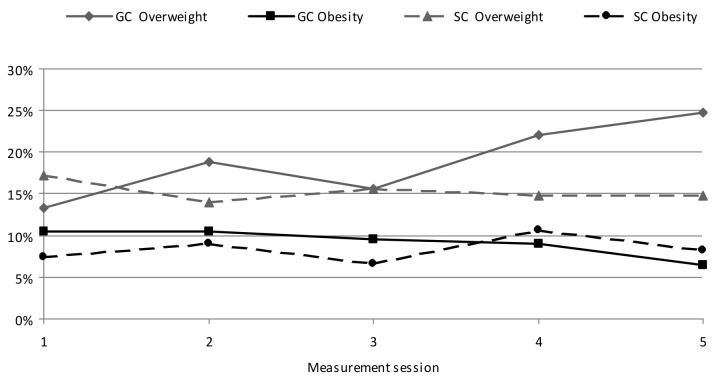
Changes in the percentage of overweight and obesity in children of standard PA (GC) and elevated PA (SC) during the course of the study.

Compared to the initial measurement session, an increase was shown in the percentage of overweight GC children by nearly 12% (from 13.31% to 24.76%; *p* = 0.260), as well as a decreased percentage of obese children by 4.03% (from 10.45% to 6.42%; *p* = 0.260) (Figure 3). In the SC, a significant decrease in the percentage of overweight children was shown (from 17.22% to 14.76%; *p* = 0.000) and 0.82% increase in the percentage of obese children (from 7.37% to 8.19%). In general, a clear downward trend was shown in the number of excessive body weight children in the SC, and a significant rising trend of overweight in the GC.

The variability of the percentage of children in particular body mass categories from the initial to the final measurement sessions are shown in Table 4. The highest differences between the GC and the SC were shown in the number of overweight children. More SC children changed the body mass category from overweight to healthy weight than GC ones (4.92% and 0.81%, respectively). Oppositely, more GC children than SC ones changed the BMI category from healthy weight to overweight (8.94% and 3.27%, respectively). Similarly, more GC children than SC ones stayed overweight (12.19% and 9.01%, respectively). This confirmed a favorable influence of elevated PA at school on children’s body mass. However, none of children, both in the GC and SC, changed body mass from obesity to healthy weight.

**Figure 4:**
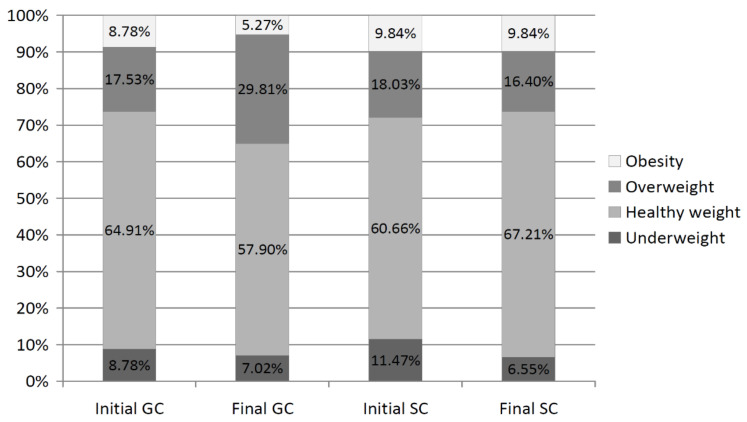
The percentage of girls in particular body mass categories—comparison between the initial and final measurement sessions.

In general, over the entire study period, nearly 65% of the children had healthy weight (GC—64.56%; SC—64.60%) (Figure 4). Overweight included 21.75% of the GC girls and 16.07% of the SC girls. Obese included 8.08% of the GC girls and 10.16% of the SC ones. In the SC girls, an increased percentage of healthy weight from 60.66% to 67.21% (*p* = 0.002) and a decreased percentage of overweight from 18.03% to 16.40% (*p* = 0.003) were observed. In the GC girls, an opposite trend was observed, i.e., a decrease in the percentage of healthy weight from 64.91% to 57.90% (*p* = 0.008) and an increase in overweight from 17.53% to 29.81% (*p* = 0.010). A decrease in the number of underweight girls was found in both the GC and the SC.

**Figure 5:**
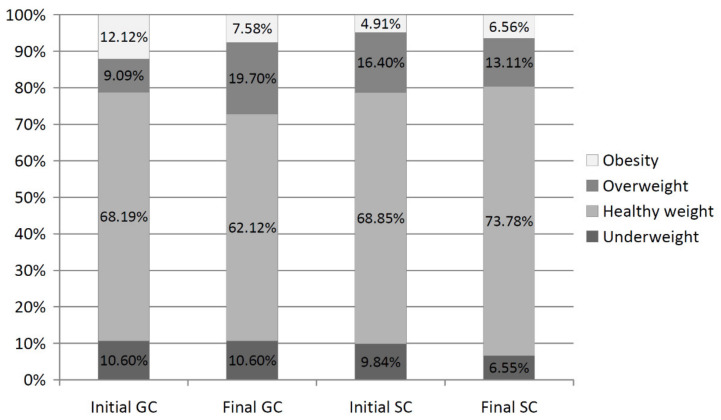
The percentage of boys in particular body mass categories—comparison between the initial and final measurement sessions.

In general, in the entire study period, nearly 67.08% of the boys had healthy weight, with a higher percentage in the SC than in the GC (GC—63.34%; SC—70.82%) (Figure 5). Underweight included 10.30% of the GC boys and 8.19% of the SC ones. During the study, a decrease in percentage of the overweight SC boys from 16.40% to 13.11% (*p* = 0.058) and an increase in healthy weight ones from 68.85% to 73.78% (*p* = 0.004) were found. In the GC, an opposite trend was observed, i.e., an increased percentage of the overweight boys from 9.09% to 19.70% (*p* = 0.000) and a decreased percentage of the healthy weight ones from 68.19% to 62.12% (*p* = 0.007).

### 3.5. PCA

**Figure 6:**
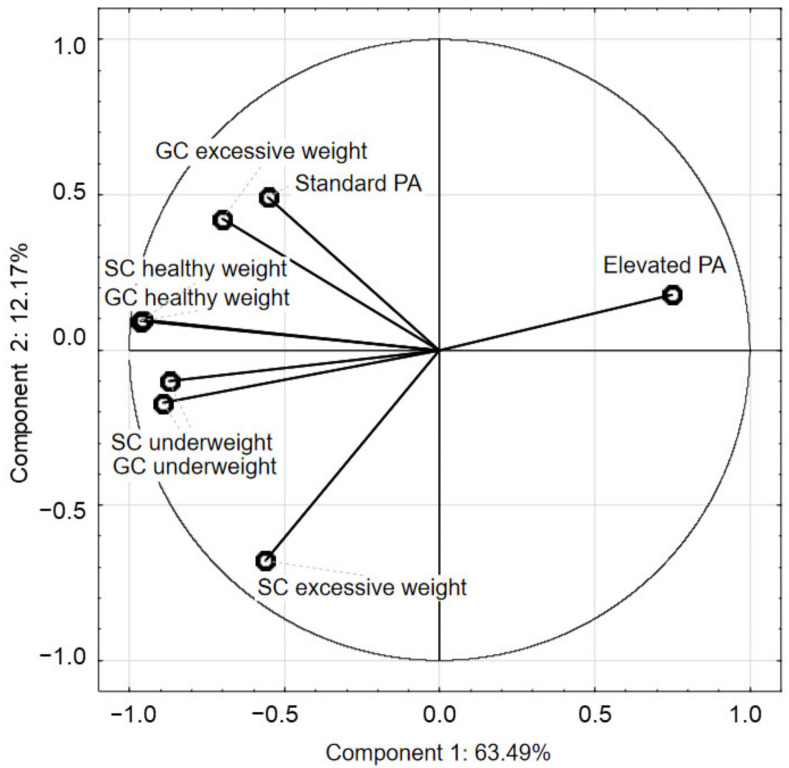
The PCA analysis of children’s body mass categories depending on the PA time at school.

PCA analysis indicated that excessive body weight (i.e., overweight and obesity) in the SC children was negatively correlated with the elevated number of PE lessons (Figure 6). However, in the GC children excessive body weight was positively correlated with standard number of PE lessons. This suggested that an elevated number of PE lessons, reflecting the time of physical activity at school, was positively associated with healthy BMI of children and led to a reduction in the risk of excessive body weight.

Table 5 shows the average BMI values for the entire study period and the standard deviation (SD) in particular body mass categories as underweight, healthy weight, and excessive body weight (overweight and obesity) in the GC and SC children. In general, the values of BMI indicating that children were underweight and overweight in both classes were generally similar, which is also shown in Figure 6. However, in the SC children, the BMI values were lower than in the GC ones (Table 5). On the other hand, the average BMI values indicating overweight were varied, and in the SC children it was a slightly lower than in the GC ones (22.65 and 23.04).

## 4. Discussion

All interventions involving an increase in PA are correct strategies to reduce the risk of overweight and obesity in children [34,35,36]. The main goal of PE lessons at school is generally to improve the physical fitness and sports activity of children, but not the only one. PE goes far beyond the sport and recreational dimension. It teaches the principles of fair play, diligence, perseverance, courage, overcoming weaknesses, ability to cooperate in a group, loyalty, and also help in the rehabilitation of young people [37]. These also apply to personal development and shaping correct social attitudes and healthy lifestyles of young people. This underlines the importance of PE lessons at school.

This study demonstrates that increased PA at school favorably influenced children’s body mass. Another important factor that impacts the risk of overweight and obesity is diet. It was demonstrated that excessive consumption of products with high energy content and irregular meal times often reduce the benefits resulting from increased time of physical activity [38,39,40].

Children aged about 10 years joined the study and participated in it up to the age of 12 years. This was because in Poland education with an elevated number of PE lessons (SC) in regular primary schools began with fourth grade of school (children age approximately 10 years). It was planned to continue the research on the same group of children until they graduated from primary school (the age of 15 years). However, the COVID-19 pandemic in 2019 forced the study to be discontinued. Therefore, at the end of the study (Sept. 2019), the children were aged 12 years. Nevertheless, in this study, a relationship between the time of physical activity at school in the form of PE lessons (type of class: general or sport) and the prevalence of excessive body weight was observed. Similar observations were made in other studies. Bi et al. (2019) demonstrated that overweight or obesity was positively related to low physical activity during adolescence [41]. In the study of Wirnitzer et al. (2021), children involved in sports activities showed a more favorable body mass compared to not involved ones [42]. Hilpert et al. (2017) showed that a low level of physical activity was the key factor affecting overweight and obesity in children [43]. Maier et al. (2013) and Godakande et al. (2018) demonstrated that the main risk factors for obesity in children were insufficient physical activity and sedentary lifestyle [44,45].

The average BMI of the children involved in this study was equal to 18.98 kg/m^2^, and was slightly higher in the GC children than in the SC ones. The BMI of the girls was equal to 18.93 kg/m^2^ and that of the boys was equal to 19.03 kg/m^2^. Another report showed that in Polish children aged 10–13 the average BMI was slightly higher and equal to 19.6 kg/m^2^ (B—19.9 kg/m^2^; G—19.3 kg/m^2^) [46]. The global BMI in children was also similar: it was equal to 18.60 kg/m^2^ for girls and 18.50 kg/m^2^ for boys [47].

This study demonstrated that in the subsequent measurement sessions, the number of overweight SC children decreased, which was not found the case of the GC ones. It was also shown that a larger number of the GC than SC children changed BMI category from initial healthy weight to overweight and larger number of the SC than GC children from initial overweight to healthy weight. Numerous studies demonstrated a higher BMI in children with a low level of physical activity [48,49]. Moreover, it was shown that overweight and obese children are much less likely to decide to undertake physical activity [50]. On average, in the entire study period, excessive body weight was observed in nearly 26% of the children, including overweight in 17.08% (G—18.91%; B—15.25%) and obesity in 8.78% (G—9.12%; B—8.43%). In relation to physical activity, overweight and obesity were noted more often in the GC children (18.90% and 9.19%, respectively) than in the SC ones (15.25% and 8.36%). In the study by Wądołowska et al. (2018), the percentage of overweight children aged 11–13 was higher than in this study and equal to over 20%, while obesity was identified in 12% of the children [51,52].

Studies by other authors confirmed that school-age boys are more involved in sport activities and games than girls, which can also have an influence on the average higher BMI in girls [53]. Moreover, the differences between the genders and the adolescence period have a clear impact on the intensity and regularity of participation in sport activities [54,55,56]. Studies by other authors confirmed that girls are, on average, less physically active than boys [57,58].

In general, the study demonstrated a constant increase in the number of overweight children in the classes of standard PA (GC) and a decrease in the classes of elevated PA (SC). In the study by Mascherini et al. (2019) overweight was observed in 22.1% of the subjects and obesity in 2.6% [56]. Other studies demonstrated a higher prevalence of overweight in girls than in boys [59,60]. However, the study by Carayanni et al. (2021) did not confirm this relationship [61].

Many reports indicated the possibility of lowering BMI through an intervention consisting in an increase in the physical activity of overweight children. However, once a child becomes obese, it is hard to achieve a long-term reduction in BMI [62,63]. The above statement conforms the observation that children identified as obese at the start of the study largely remained obese at the end of the study. After starting education in classes with an elevated PA, a higher number of children changed body mass category from excessive weight to healthy weight, while for children with the standard PA a change from obesity to overweight or healthy weight was observed much more rarely.

The results of this study confirmed that an increase in children’s physical activity at school brings a desirable health effect. Additional, extra-curricular physical activity can be a very effective way to reduce the risk of obesity in school-age children.

Despite demonstration of significant trends, the study presented herein has certain limitations. The study covered only 2 years of observation. It was planned to continue the research on the same group of children until they graduated from primary school (8th class, 15 years). However, the outbreak of the COVID-19 pandemic and the associated frequent lockdowns and online education resulted in discontinuation of the study. Moreover, the study was limited only to a relatively small sample of children between the ages of 10 and 12 from Siedlce. It is worth extending the research to children in other age groups and from larger areas. Another limitation is that body mass index is influenced not only by physical activity level but also by nutritional behavior and socioeconomic status, however, the analysis did not take this into account. It is advisable to extend the research by including the assessment of nutritional behavior, as well as body fat and muscle tissues content, in order to more widely demonstrate the effect of elevated physical activity on children’s health.

The strong point of the study was the selection of groups of children with increased and standard physical activity at school (10 and 4 h PE per week, respectively), with very similar number of participants (122 and 123, respectively). Another strong point was a research team that was qualified in conducting and interpreting the research. This ruled out the risk of errors in this regard. In addition, the analysis covered only children who participated in all measurement sessions, which increased the accuracy of the results.

## 5. Conclusions

Starting from the similar level, during the study the percentage of excessive body weight (overweight and obese) increased by ¼ in children of standard PA at school while slightly decreased in children of elevated PA. The body mass of children after two years of elevated PA became healthier than those of standard PA. The beneficial effect of elevated PA at school on the children’s body mass was shown. A much higher number of elevated PA children changed body mass category from initial overweight to healthy weight than those of standard PA. Girls, especially of standard PA, had more often excessive body weight compared to boys. Increasing time of physical activity at school by elevation of the number of PE lessons favorably affects the body mass of children.

## Figures and Tables

**Table 1:** The percentage of body mass categories in the GC and SC children during the course of the study.

Variables	Measurement Session									
	I		II		III		IV		V	
Average age (years)	10.27		10.90		11.27		11.90		12.26	
Class type	GC	SC	GC	SC	GC	SC	GC	SC	GC	SC
Underweight	9.69	10.66	6.30	9.83	9.45	9.84	5.54	6.55	8.81	6.55
Healthy weight	66.55	64.75	64.39	67.22	65.39	68.03	63.40	68,04	60.01	70.51
Overweight	13.31	17.22	18.86	13.94	15.59	15.57	22.01	14.75	24.76	14.76
Obesity	10.45	7.37	10.45	9.01	9.57	6.56	9.05	10.66	6.42	8.19

GC—general education classes (standard PA); SC—sport classes (elevated PA); measurement sessions: I—September 2017; II—March 2018; III—September 2018; IV—March 2019; V—September 2019.

**Table 2:** The percentage of body mass categories in the GC and SC girls during the course of the study.

Variables	Measurement Session									
	I		II		III		IV		V	
Average age (years)	10.27		10.90		11.27		11.90		12.26	
Class type	GC	SC	GC	SC	GC	SC	GC	SC	GC	SC
Underweight	8.78	11.47	3.50	11.47	5.26	9.84	3.51	6.55	7.02	6.55
Healthy weight	64.91	60.66	66.67	62.31	70.18	67.21	63.16	65.57	57.90	67.21
Overweight	17.53	18.03	21.05	14.75	17.54	14.75	22.81	16.40	29.81	16.40
Obesity	8.78	9.84	8.78	11.47	7.02	8.20	10.52	11.48	5.27	9.84

GC—general education classes (standard PA); SC—sport classes (elevated PA); measurement session: I—September 2017; II—March 2018; III—September 2018; IV—March 2019; V—September 2019.

**Table 3:** The percentage of body mass categories in the GC and SC boys during the course of the study.

Variables	Measurement Session									
	I		II		III		IV		V	
Average age (years)	10.27		10.90		11.27		11.90		12.26	
Class type	GC	SC	GC	SC	GC	SC	GC	SC	GC	SC
Underweight	10.60	9.84	9.09	8.19	13.64	9.84	7.57	6.55	10.60	6.55
Healthy weight	68.19	68.85	62.12	72.13	60.60	68.85	63.64	70.50	62.12	73.78
Overweight	9.09	16.40	16.67	13.12	13.64	16.39	21.22	13.11	19.70	13.11
Obesity	12.12	4.91	12.12	6.56	12.12	4.92	7.57	9.84	7.58	6.56

GC—general education classes (standard PA); SC—sport classes (elevated PA); measurement session: I—September 2017; II—March 2018; III—September 2018; IV—March 2019; V—September 2019.

**Table 4:** The variability of the percentage of children in particular body mass categories from the initial to the final measurement sessions.

Variables	GC	SC
	Underweight	
Initial measurement session	9.69%	10.66%
Final measurement session	8.81%	6.55%
stayed underweight	5.69%	5.74%
from underweight to healthy weight	4.06%	4.92%
from healthy weight to underweight	3.12%	0.82%
Overweight		
Initial measurement session	13.31%	17.22%
Final measurement session	24.74%	14.76%
stayed overweight	12.19%	9.01%
from overweight to healthy weight	0.81%	4.92%
from healthy weight to overweight	8.94%	3.27%
from overweight to obesity	0.81%	2.50%
from obesity to overweight	3.61%	2.48%
Obesity		
Initial measurement session	10.45%	7.37%
Final measurement session	6.42%	8.19%
stayed overweight	5.61%	5.69%
from overweight to obesity	0.81%	2.50%
from obesity to healthy weight	none	none
from healthy weight to obesity	none	none

GC—general education classes (standard PA); SC—sport classes (elevated PA).

**Table 5:** The average BMI values of children in particular body mass categories during the entire study period.

Body Mass Categories	Average BMI (kg/m^2^)	SD
GC underweight	14.33	0.48
GC healthy weight	16.08	0.43
GC excessive weight	23.04	2.18
SC underweight	14.04	0.41
SC healthy weight	16.27	0.71
SC excessive weight	22.65	1.28

SD—standard deviation; GC—general education classes (standard PA); SC—sport classes (elevated PA).

## Data Availability

Data is available upon request.
